# Complex Evolution of a Y-Chromosomal Double Homeobox 4 (DUX4)-Related Gene Family in Hominoids

**DOI:** 10.1371/journal.pone.0005288

**Published:** 2009-04-30

**Authors:** Julia Schmidt, Stefan Kirsch, Gudrun A. Rappold, Werner Schempp

**Affiliations:** 1 Institute of Human Genetics, University of Freiburg, Freiburg, Germany; 2 Institute of Human Genetics, University of Heidelberg, Heidelberg, Germany; Mount Sinai School of Medicine, United States of America

## Abstract

The human Y chromosome carries four human Y-chromosomal euchromatin/heterochromatin transition regions, all of which are characterized by the presence of interchromosomal segmental duplications. The Yq11.1/Yq11.21 transition region harbours a peculiar segment composed of an imperfectly organized tandem-repeat structure encoding four members of the double homeobox (DUX) gene family. By comparative fluorescence in situ hybridization (FISH) analysis we have documented the primary appearance of Y-chromosomal DUX genes (DUXY) on the gibbon Y chromosome. The major amplification and dispersal of DUXY paralogs occurred after the gibbon and hominid lineages had diverged. Orthologous DUXY loci of human and chimpanzee show a highly similar structural organization. Sequence alignment survey, phylogenetic reconstruction and recombination detection analyses of human and chimpanzee DUXY genes revealed the existence of all copies in a common ancestor. Comparative analysis of the circumjacent beta-satellites indicated that DUXY genes and beta-satellites evolved in concert. However, evolutionary forces acting on DUXY genes may have induced amino acid sequence differences in the orthologous chimpanzee and human DUXY open reading frames (ORFs). The acquisition of complete ORFs in human copies might relate to evolutionary advantageous functions indicating neo-functionalization. We propose an evolutionary scenario in which an ancestral tandem array DUX gene cassette transposed to the hominoid Y chromosome followed by lineage-specific chromosomal rearrangements paved the way for a species-specific evolution of the Y-chromosomal members of a large highly diverged homeobox gene family.

## Introduction

Among human chromosomes the Y chromosome shows the highest proportion of segmental duplications [1]–[3], a class of low-copy repeats implicated in the large-scale variation of the human genome [4]. Such duplicated sequences are found interspersed throughout the genome, however they predominantly tend to cluster in pericentromeric and subtelomeric regions [4]–[6]. Not surprisingly all four Y-chromosomal euchromatin/heterochromatin transition regions are composed of duplicated sequences [7], [8]. Within the Yq11.1/Yq11.21 transition region a specific genomic segment of ∼30 kb is framed by segmental duplications, but presents distinctive differences to its direct genomic environment. This segment is characterized by the presence of an imperfectly organized tandem-repeat structure encoding members of the DUX gene family [8]. Recurrent alternating repeat elements of the LSAU and 68 bp beta-satellite repeat family form a scaffold in which the DUX genes are embedded. Length variations of the tandem repeat are exclusively restricted to the beta-satellite regions.

This basic structure is highly similar to the architectural features of the D4Z4 tandem array, a 3.3 kb repeat unit located in highly variable numbers in 4q35 [9], [10]. The polymorphic repeat also encodes a member of the DUX gene family [11], [12] termed *DUX4* which is supposed to have a major impact on the etiology of FSHD (Facioscapulohumeral muscular dystrophy;[13]–[15]), the third most common muscular dystrophy [16]. Although a similar tandem array exists in 10q26, no association with FSHD could be established [17]–[19]. Additional copies of the DUX gene family with nucleotide sequence identities ranging from 80–99% are scattered throughout the heterochromatic regions of the short arms of all acrocentric chromosomes and chromosomal bands 1q12, 9q12, and 10cen [10]. Due to their unusual organization and chromosomal distribution pattern DUX-containing repeats reflect a specific category of segmental duplications.

Recently, strong evidence has been provided that the DUX4 open reading frame is evolutionarily conserved. Homologues were identified in the genomes of rodents, Afrotheria and several other species. Moreover, phylogenetic analysis discloses the descent of the primate and Afrotherian *DUX4* orthologs from a retrotransposed copy of an intron-containing DUX gene [20]. Although this study profited from the extensive whole-genome sequence data, no proof was provided of the existence of Y-chromosomal DUX copies in non-human primates. This can be easily explained by the obvious preference for female individuals in such efforts.

Here we focus on the evolutionary history of DUX genes on the primate Y chromosomes. The date of initial appearance of Y-chromosomal DUX copies is documented and species-specific varying Y-chromosomal localizations are identified. Furthermore, the autosomal distribution pattern of DUXY-related genes provides evidence for their enormous increase in dispersal and amplification in the higher primate genome. Detailed comparative analysis of the human and common chimpanzee DUXY locus allowed us to infer the evolutionary processes shaping its basic structural organization.

## Results

### Cosmid contig of the human DUXY locus

**Figure 1:**
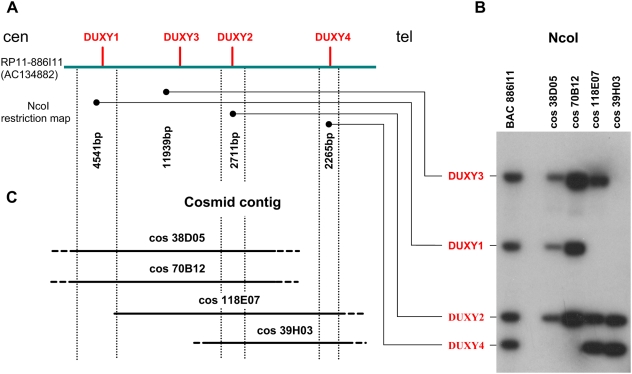
Chromosomal organization of the human Y-chromosomal DUX locus with respect to the centromere-telomere orientation. *(A)*. The bar labeled on the left side with RP11-886I11 (AC134882 [8]) comprises the complete human DUXY locus. Only the BAC section harboring the locus is shown. The position of each DUXY gene is indicated by a red vertical line. Vertical dotted lines depict NcoI restriction sites. Each DUXY gene is represented by a characteristic restriction fragment. The size of each fragment is given below the corresponding DUXY gene. *(B)*. Southern blot hybridization of the 281-bp DUXY PCR fragment to NcoI-digested DUXY BAC and cosmid DNAs. Along the left the DUXY genes giving rise to each fragment are listed. According to the hybridizing fragments the overlap of the DUXY cosmids can be determined *(C)*. Cosmid contig of the human DUXY locus. Cosmids are positioned along the NcoI restriction map with respect to their DUXY content. The library address of each cosmid is indicated.

By filter hybridization of the human Y chromosome-specific cosmid library LL0YNC03″M″ we identified 36 DUXY-positive cosmid clones, of which 22 were further analyzed. Using the NcoI restriction map of BAC clone RP11-886I11 (AC134882) as a framework (Figure 1A), we determined the DUXY gene content of each cosmid. Five cosmids were positive for *DUXY1*, three for *DUXY1* and *DUXY3*, two for *DUXY1*, *DUXY2*, and *DUXY3*, two for *DUXY3*, *DUXY2*, and *DUXY4*, three for *DUXY2* and *DUXY4* and seven for *DUXY4*. None of the cosmids contained all four DUXY genes. The DUXY gene content and relative position of four selected cosmids (LL0YNC03″M″-38D05, -39H03, -70B12, and -118E07) are illustrated in Figure 1B and C. Cosmids LL0YNC03″M″-38D05 and -70B12 contain *DUXY1–3* and show identical restriction patterns (data not shown). Cosmids LL0YNC03″M″-118E07 and -39H03 contain *DUXY2–4,* and *DUXY2* and *DUXY4,* respectively.

Given the average insert size of a cosmid and the genomic extension of the DUXY locus (30.3 kb; [8]) it is obvious that all cosmid clones carry sequences in addition to the actual DUXY locus. Towards the centromere a stretch of 14 kb of satellite sequences and other repeats is adjacent to the DUXY locus whereas towards the telomere segmental duplications are attached. Therefore cosmids LL0YNC03″M″-70B12 and -38D05 exclusively detect *DUXY*-specific sequences and their paralogs. In addition, cosmids LL0YNC03″M″-118E07 and -39H03 identify paralogs of the segmental duplications bordering the distal boundary of the DUXY locus.

### Comparative FISH of DUXY cosmids on the Y chromosome of human and non-human primates

**Figure 2:**
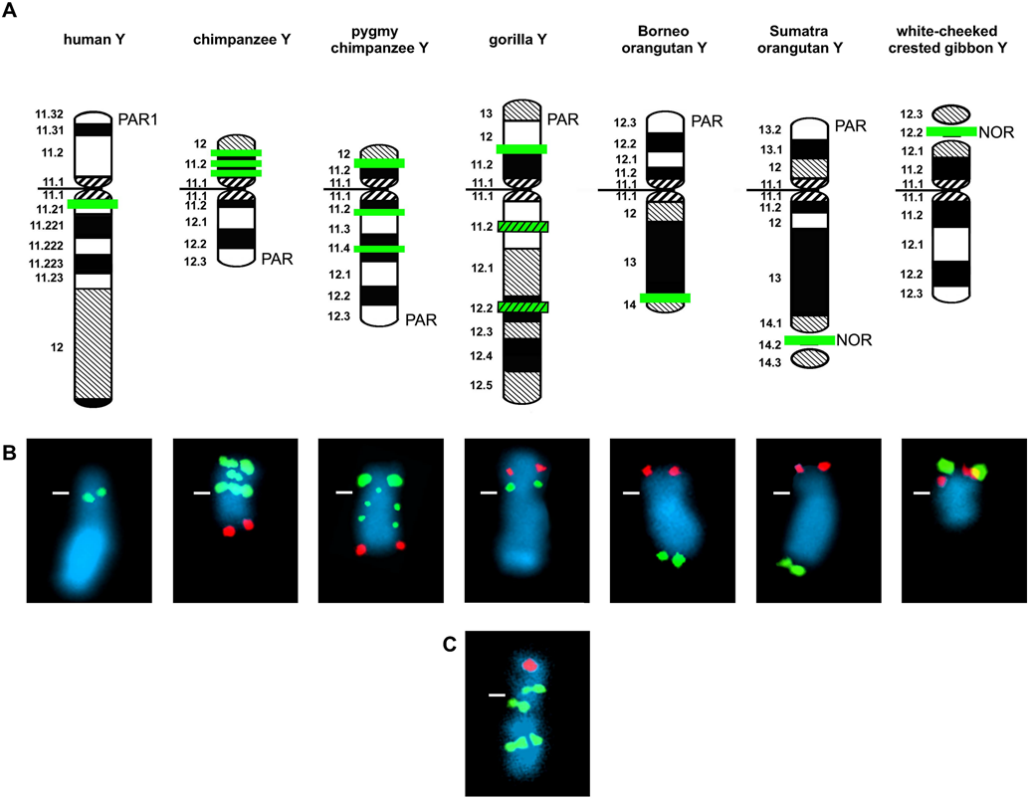
Overview of the species-specific Y-chromosomal locations of DUXY genes in hominoids. Comparative FISH analysis was performed with human DUXY-containing cosmids LL0YNC03″M″-39H03, -70B12, and -118E07. *(A)* For each Y chromosome analyzed a G-banded ideogram is outlined. The position of the pseudoautosomal regions (PAR1 in human; PAR in great apes) and the nucleolus organizer region (NOR) is depicted on the respective ideograms. The cytogenetic location of DUXY genes is indicated as a green horizontal bar. Please note that the gorilla Y chromosome presents a distinctive difference in the hybridization pattern of cosmids 39H03 and 118E07 (standard green bar) when compared with cosmid 70B12 (standard plus striped green bar). *(B)* FISH of cosmid LL0YNC03″M″-39H03 (green signals) on DAPI-counterstained metaphase Y chromosomes of hominoid species. For the ease of chromosomal orientation, the human pseudoautosomal cosmids ICRFc104E0238 (IL3RA [59]) or LL0YNC03″M″-34F05 (SHOX [60]) were co-hybridized in great apes and the mouse rDNA-containing plasmid pMR100 [61] in gibbon (red signals). The position of the centromere is indicated as a white horizontal line. *(C)* Distinctive FISH pattern of cosmid LL0YNC03″M″-70B12 on a metaphase Y chromosome of the gorilla.

To investigate the evolutionary history of the human Y-chromosomal DUX locus, we comparatively mapped the DUXY-containing cosmids LL0YNC03″M″-70B12, -118E07, and -39H03 on metaphase chromosomes from human and non-human primates. A summary of the mapping data on the Y chromosomes is depicted in Figure 2. On the human Y chromosome all cosmids hybridize consistently to the Yq11.2/Yq11.21 transition region. With the exception of the gorilla Y chromosome all cosmids also show identical signal patterns on the Y chromosomes of the non-human primates. Nevertheless, the signal pattern is quite specific for each species. On the common chimpanzee Y chromosome, three equally intense signals are seen in proximal, central and distal Yp11.2. Only the distal signal coincides in intensity and location to a signal on the pygmy chimpanzee Y chromosome. Two fainter signals on the pygmy chimpanzee Y chromosome map to the proximal (Yq11.2/Yq11.3) and central part (Yq11.4) of the long arm. On the gorilla Y chromosome, all cosmids show one signal in the central part of the short arm (Yp11.3/Yp12), but cosmid LL0YNC03″M″-70B12 additionally detects two locations in the long arm (Yq11.2; Yq12.2). In both orangutan subspecies and the white-cheeked crested gibbon all cosmids detect only one signal. In the Bornean orangutan the signal is situated at the boundary to the heterochromatic cap of the long arm (Yq13/Yq14), whereas in the Sumatran orangutan and the white-cheeked crested gibbon the signal maps to the nucleolus organizer region (NOR). None of the cosmids showed signals on the rhesus macaque Y chromosome.

### Comparative FISH analysis of autosomal paralogies of DUXY cosmids

**Figure 3:**
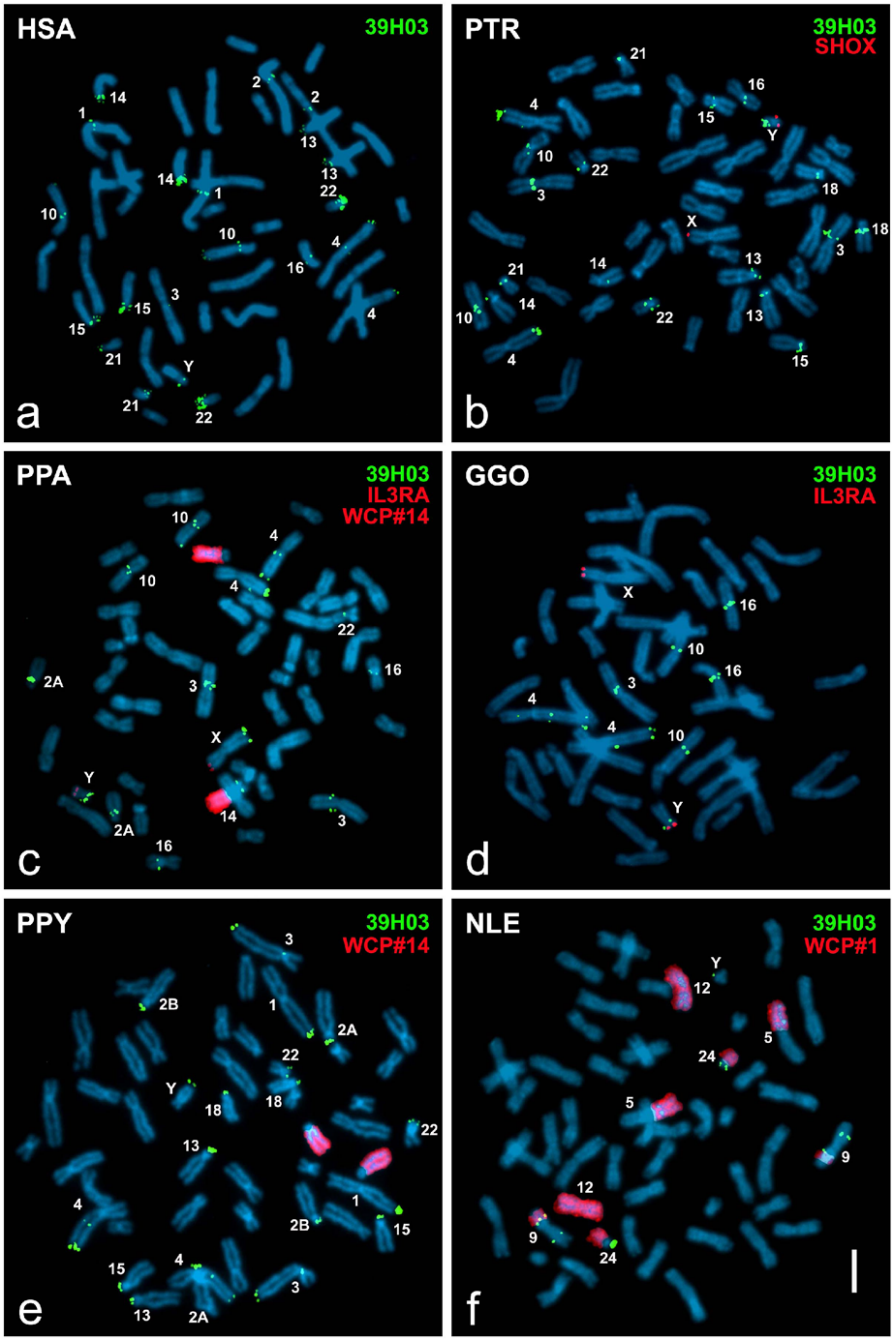
Comparative FISH of human Y-chromosomal DUXY-containing cosmid LL0YNC03″M″-39H03 on human and non-human primate male individuals. The signal pattern (green) is shown on human (HSA), common chimpanzee (PTR), pygmy chimpanzee (PPA), gorilla (GGO), Sumatra orangutan (PPY), and white-cheeked crested gibbon (NLE) metaphase spreads. Cosmids ICRFc104E0238 [59] or LL0YNC03″M″-34F05 [60] were used as a marker for the pseudoautosomal region (red) on the distal long arm of both chimpanzee Y chromosomes and the gorilla Y chromosome (red); human probe WCP#14 tagged chromosome 14 orthologs in pygmy chimpanzee and orang-utan (red) and WCP#1 allowed the allocation of signal positions on NLE chromosomes (red).

The paralogous multi-site pattern on the autosomes was documented for the species analyzed on the chromosomal band level (Table 1). Autosomal paralogies were detected after hybridization of the cosmids in all ape species investigated. The multi-site signal pattern of LL0YNC03″M″-39H03 on metaphase spreads of the human, common and pygmy chimpanzee, gorilla, Bornean orangutan, and white-cheeked crested gibbon is illustrated in Figure 3. The most prominent difference in the multi-site signal patterns of cosmid LL0YNC03″M″-70B12 and cosmids LL0YNC03″M″-118E07/-39H03 seen in human is the signal in 4q24 (Figure 3a). This finding can easily be explained by the presence of a duplicon in the latter cosmids whose ancestral state was shown to reside in this chromosomal region [21]. Signals were detected in the orthologous positions of all non-human primates (4q24 in the great apes and 9qmed in the gibbon), too. Moreover, cosmid LL0YNC03″M″-70B12 causes strong signals in 9cen and 9p11.2, whereas cosmid LL0YNC03″M″-118E07 shows weak and polymorphic signals at the same chromosomal locations and cosmid LL0YNC03″M″-39H03 presents no signals at all on chromosome 9 (data not shown). In both chimpanzee species, both orangutan subspecies and the white-cheeked crested gibbon, the multi-site signal patterns detected by each cosmid are consistent. However, in the gorilla distinct quantitative signal differences with cosmid LL0YNC03″M″-70B12 were noted (Table 1). Six additional chromosomal locations (2B, 9, 13, 14, 15, 18) are solely detected by this cosmid. Comparable with the macaque Y chromosome, none of the cosmids show signals on the macaque autosomes.

**Table 1:** Comparative FISH results for human DUXY-containing cosmids.

Clone	HSA	PTR	PPA	GGO	PPY	NLE
LLNLY-70B12	1q12; 2cen; 3cen; 4q35; 9p11.2; 9cen; 9q11.2; 10p11.2; 10q11.2; 10q26; 13*; 14*; 15*; 16cen; 21*; 22*	3p13; 4q15; 4q25; 10p11.2; 10q11.2; 13*; 14*; 15*; 16p13; 18*; 21*; 22*	2Acen; 3p13; 4q15; 4q25; 10p11.2; 10q11.2; 10q26; 14cen; 16p13; 22cen;	2Bcen; 3p13; 4q25; 9p11.2; 9cen; 10cen; 13*; 14*; 15*; 16p13; 16cen; 18*	1q36; 2Acen; 2Bcen; 3cen; 3q39; 4q15; 4q25; 13*; 15*; 18*; 22*	9pmed; 9qmed; 24p
LLNLY-118E07	1q12; 2cen; 3cen; 4q24; 4q35; 9cen; 10p11.2; 10q11.2; 10q26; 13*; 14*; 15*; 16cen; 21*; 22*	3p13; 4q15; 4q25; 10p11.2; 10q11.2; 13*; 14*; 15*; 16p13; 18*; 21*; 22*	2Acen; 3p13; 4q15; 4q25; 10p11.2; 10q11.2; 10q26; 14cen; 16p13; 22cen;	3p13; 4p13; 4q15; 4q25; 10cen; 16p13; 16cen;	1q36; 2Acen; 2Bcen; 3cen; 3q39; 4q15; 4q25; 13*; 15*; 18*; 22*	9pmed; 9qmed; 24p
LLNLY-39H03	1q12; 2cen; 3cen; 4q24; 4q35; 10p11.2; 10q11.2; 10q26; 13*; 14*; 15*; 16cen; 21*; 22*	3p13; 4q15; 4q25; 10p11.2; 10q11.2; 13*; 14*; 15*; 16p13; 18*; 21*; 22*	2Acen; 3p13; 4q15; 4q25; 10p11.2; 10q11.2; 10q26; 14cen; 16p13; 22cen;	3p13; 4p13; 4q15; 4q25; 10cen; 16p13; 16cen;	1q36; 2Acen; 2Bcen; 3cen; 3q39; 4q15; 4q25; 13*; 15*; 18*; 22*	9pmed; 9qmed; 24p

DUXY-containing cosmids were hybridized to metaphase spreads of human and non-human primate species. FISH results are listed for HSA (*H. sapiens*), PTR (*P. troglodytes*), PPA (*P. paniscus*), GGO (*G. gorilla*), PPY (*P. pygmaeus*), and NLE (*N. leucogenys*). Broad signals comprising the centromere and short arm of an acrocentric chromosome are indicated with an asterisk (*). The chromosomal designations for the great apes are given according to the human phylogenetic group [81], for NLE according to [82].

Taken together, the comparative analysis of the multi-site signal patterns in human and the great apes shows signals present at orthologous locations of several species (Table 1, Figure 3). In contrast, the signal in human 1q12 (Figure 3a) has no corresponding orthologous signal in the great apes. A minimum of two short arms of acrocentric chromosome pairs is labeled in each great ape species. Yet, in the human and the common chimpanzee all acrocentric chromosome pairs show clear signals (Figure 3a,b). Moreover, some signals are restricted to one species, e.g. only the orangutan shows signals in 1p36 and 3q39 (Figure 3e). The signal uniquely visible in the distal long arm of the pygmy chimpanzee X chromosome (Figure 3c) reflects a genomic variant, as it was detected exclusively in this individual.

### Comparative sequence analysis of the human and chimpanzee DUXY locus

#### Comparative genomic organization of the DUXY loci

**Figure 4:**
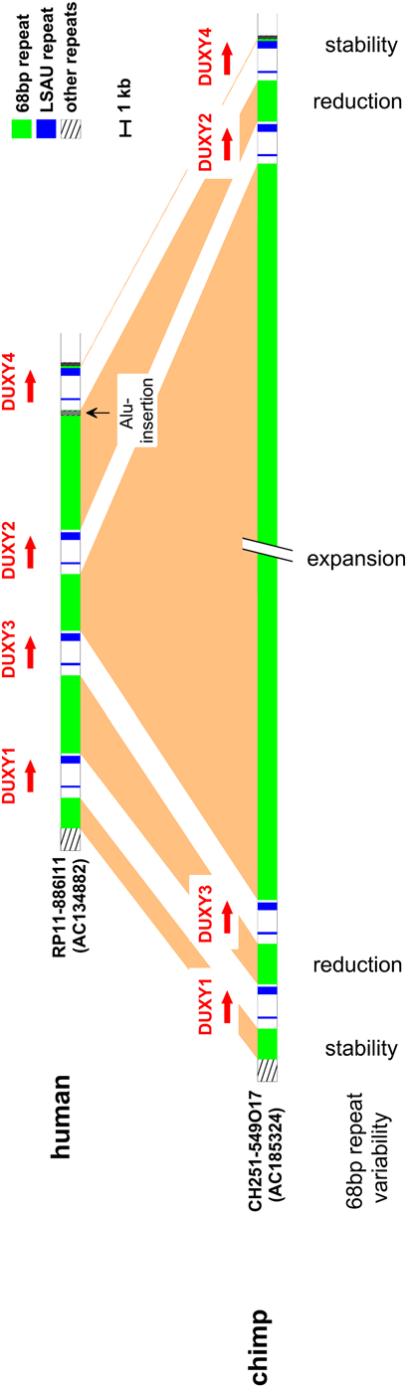
Comparative illustration of the sequential composition of the human and chimpanzee DUXY locus. The basic structural organization of the chimpanzee DUXY locus is strikingly similar to that of the human DUXY locus [8]. Orthologous DUXY gene copies (*DUXY1–4*) are found at orthologous locations within both DUXY loci and the transcription orientation (indicated as a red arrow) is preserved. In each species DUXY genes are a basic component of an imperfect tandem repeat consisting of a beta-satellite (basic monomer of 68 bp; green) and an LSAU repeat (blue). The enormous difference in DUXY locus length is caused by a major expansion of one chimpanzee beta-satellite block (3884 bp in human↔145021 bp in chimpanzee). All other orthologous beta-satellite repeat blocks show more moderate or only small size differences (1999 bp↔2007 bp; 5243 bp↔2905 bp; 7709 bp↔2866 bp; 125 bp↔135 bp). Both blocks of LSAU repeats are quite constant in size. Human and chimpanzee sequences enclosing DUXY loci are also conserved. The *Alu* insertion noted in the human DUXY locus is human-specific.

To better understand the evolution of the DUXY locus in primates, we investigated the basic structural organization of the orthologous locus on the chimpanzee Y chromosome. In human, we previously identified and sequenced a Y-chromosomal BAC clone [RP11-886I11 (AC134882)] containing the complete human DUXY locus [8]. To compare the genomic structure of the human DUXY locus to that of the common chimpanzee, we identified by a BLAST sequence similarity search against GenBank a completely sequenced Y-chromosomal chimpanzee BAC clone [CH251-549O17 (AC185324)] covering the entire orthologous DUXY locus. The basic genomic properties of both DUXY loci are depicted in Figure 4.

The orientations and sizes of all 4 DUXY genes of the human and the common chimpanzee were the same, whereas the genomic extension of the complete DUXY locus in the chimpanzee was 134.6 kb longer than that in human. This difference in size results solely from the length variability of the beta-satellite repeat blocks. Whereas in four out of five repeat blocks in the chimpanzee DUXY locus a moderate reduction or stability in overall length is noted, the repeat block in the center of the chimpanzee DUXY locus has experienced a major expansion (human: 3884 bp↔chimp: 145021 bp). Moreover, it is noteworthy that the repeat blocks marking the boundaries of the human and chimpanzee DUXY locus are stable in size. At the transition region from the repeat block to the 5′-site of *DUXY4* a human-specific Alu-insertion was identified.

Three further incompletely sequenced chimpanzee Y-chromosomal BAC clones [CH251-270L13 (AC185326); CH251-179K02 (AC196580); CH251-399P14 (AC198668)] were also found to contain significant nucleotide sequence identities to the human DUXY copies. Owing to their highly fragmented draft sequence status and the complex structure of the DUXY locus we omitted these BAC clones from sequential analyses.

#### Comparative analysis of monomeric beta-satellites

To explore the evolutionary relationships among the human and chimpanzee beta-satellite repeat blocks, we broke all ten repeat blocks (Roman numerals I–V with respect to the centromere-telomere orientation in human) into basic ∼68 bp monomers. CLUSTALW alignments [22] between all possible pairwise combinations of the 2506 monomers were used to graphically illustrate the relationships between monomers (data not shown). Detailed versions of percent identity scores from the corresponding human and chimpanzee beta-satellite repeat blocks I, II, and IV are displayed in Figure S1. Within these beta-satellite blocks the monomer percent identity ranged from ∼69% to ∼75% (Table 2). A detailed analysis of beta-satellite repeat blocks III may be found in Text S1 and Figure S2. The highest degree of sequence conservation of monomeric beta-satellites among both species was always found between the two repeat blocks in orthologous position within the DUXY locus. Interestingly, in all beta-satellite repeat blocks the mean percent identity of the monomers is, on average, 3.6% higher in human than in chimpanzee.

**Table 2:** Evolutionary relationship among monomers from human and cimpanzee beta-satellite regions.

beta-satellite regions	human		chimp		human vs. chimp	
	Number of monomers	sequence identity (%)	Number of monomers	sequence identity (%)	Number of monomers	sequence divergence (%)
I	28	72,3	29	68,8	28	7,2
II	74	74,6	41	71,5	38	4,6
III	56	73,4	2121	80,9 (70,6)*	43	7,3
			1429 (HOR102)†	98,4		
			677 (HOR45)†	73,5		
IV	110	74,8	41	69,6	40	4,4
V	1	-	1	-	1	1,5

* The percent identity value in parentheses indicates the calculated monomer sequence identity in beta-satellite region III of the chimp after substituting all multimers of both HOR arrays by a consensus multimer each.

† The HOR arrays are named according to their multimer number.

To address the amount of sequence conservation of monomeric beta-satellites between human and chimpanzee, we determined the exact location of the orthologous beta-satellite stretches within each beta-satellite repeat block. We compared 150 aligned orthologous monomers from the DUXY locus of both species and found a sequence divergence of 5.8% (Table 2), higher than the sequence divergence between the human and chimpanzee Y chromosome (1.72% [23]; 1.78% [24]). The sequence divergence of these monomer orthologs corresponds approximately with the sequence divergence between the human and chimpanzee centromeric alpha-satellite higher-order repeat (HOR) on the X chromosome (7.0% [25]), but is significantly higher than the sequence divergence between the human and chimpanzee centromeric alpha-satellite HOR on the Y chromosome (20%; determined from the CLUSTALW alignment of the human Y chromosome major repeat {DYZ3 [26]} and the chimpanzee Y chromosome major repeat {ALRY-MAJOR_PT [27]}).

#### Comparative analysis of the DUXY genes

**Figure 5:**
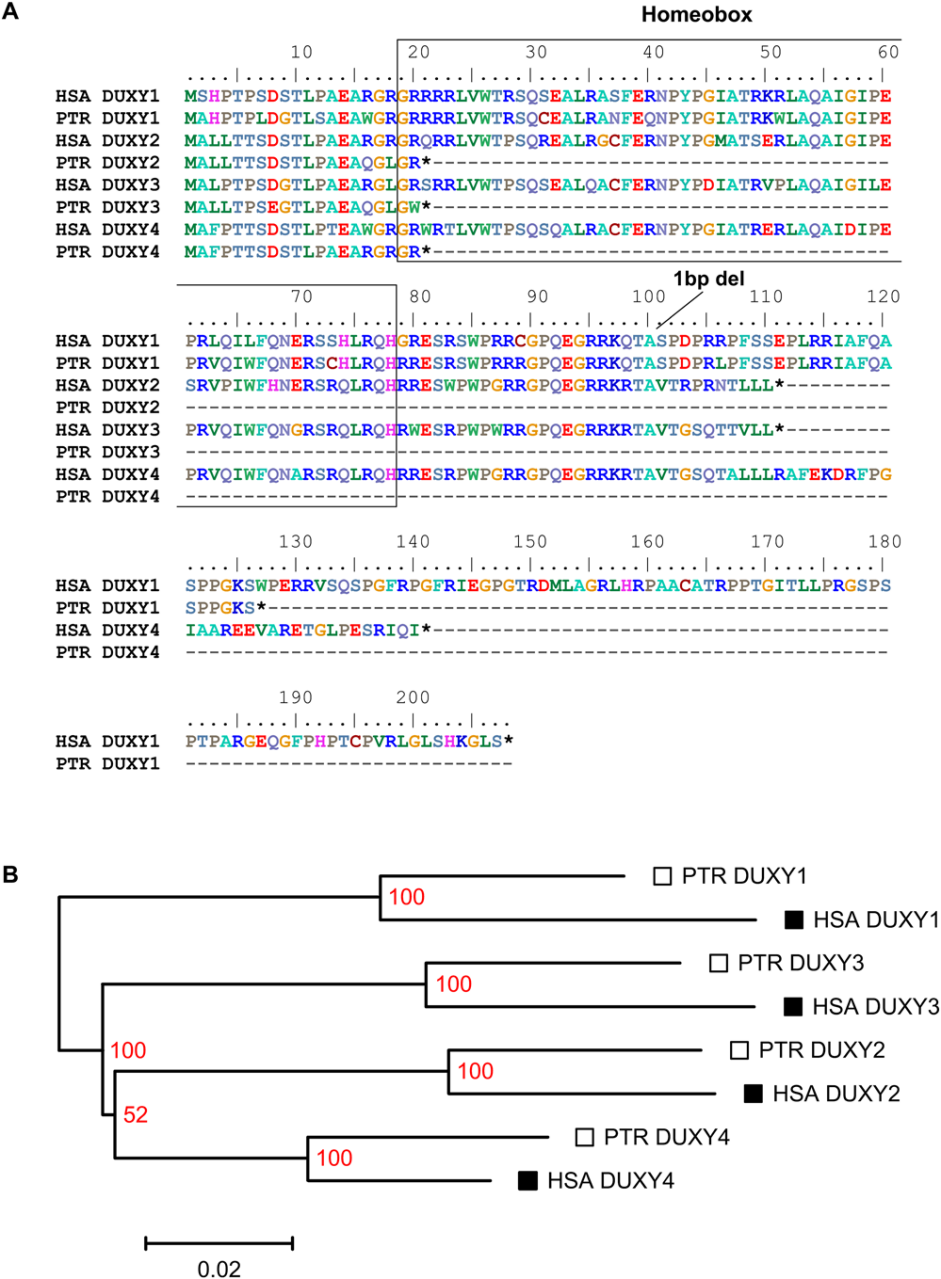
Amino acid sequence comparison and phylogenetic relationships of human and chimpanzee DUXY copies. *(A)* Comparison of the predicted amino acid sequence of orthologous human (HSA DUXY1–4) and chimpanzee (PTR DUXY1–4) Y-chromosomal DUX copies. The colour code corresponds to the CLUSTALW default for amino acid sequence alignments. Analogous to the human DUXY genes, none of their chimpanzee counterparts contain a second homeodomain. The 1-bp deletion previously identified in HSA DUXY1 is also present in PTR DUXY1, the only chimpanzee DUXY gene copy presumably capable to encode a functional protein. The chimpanzee DUXY copies *DUXY2–4* carry a stop codon at position 21 of the putative DUXY open reading frame. *(B)* Phylogenetic analysis of DUXY gene copies of human and chimpanzee. The gene phylogeny is based on a molecular phylogenetic analysis using maximum likelihood methods. Topology and branch lengths were generated with TreeView. Each branch termini is labeled with the abbreviation of the corresponding DUX gene copy. The bootstrap value is positioned without any spacing at the branching point (red).

The four Y-chromosomal copies of the human DUX gene family (*DUXY1–4*) are predicted to encode ORFs ≥110 amino acids including the first homeodomain [8]. Direct comparison of the predicted human amino acid sequences to those of the four chimpanzee DUXY copies revealed that only one chimpanzee copy (PTR *DUXY1*) retained the ability to encode a homeodomain (Figure 5A). The chimpanzee *DUXY* copies *2–4* have all experienced a C→T transition at the first position of codon 21 creating a stop mutation (TGA). Interestingly, in human the sequence of codon 21 is different in all 4 DUXY copies.

To investigate the substitution rates in the putative ORFs of the DUXY1 orthologs, we determined the number of nonsynonymous (*d*_N_) and synonymous substitutions (*d*_S_). A total of 15 nonsynonymous and 7 synonymous substitutions were detected, thus yielding a *d*_N_/*d*_S_ of 2.14. The majority of the nonsynonymous substitutions were found in the amino terminus. Five of the first 16 amino acids in PTR DUXY1 are altered in HSA DUXY1. Only one nonsynonymous substitution was detected in the carboxy terminus specific to the DUXY1 orthologs. The short stretch of preserved amino acid sequence in three of four chimpanzee DUXY copies prevented identical tests on the DUXY2–4 orthologs.

For the same reason that applies to the previous tests on DUXY2–4 orthologs, maximum likelihood estimations of amino acid phylogenies yielded only weak bootstrap support (bootstrap values of 36–61%) for the internal nodes in the phylogenetic tree. We therefore compared the corresponding nucleotide sequences of all human and chimpanzee DUXY copies as delimited by the ORF of HSA DUXY1 on the genomic level. The alignment was of high quality over the entire region of each sequence. The maximum likelihood-based phylogenetic tree from all eight genes is displayed in Figure 5B. Except for the *DUXY2–4* branching point all internal nodes are supported by bootstrap analysis. The resulting tree topology is in good agreement with the comparative analysis of monomeric beta-satellites confirming the existence of the basic DUXY locus structure in an ancestor of human and chimpanzee.

To exclude if species-specific recombination events affected the phylogenetic reconstruction, we conducted a recombination signal analysis with the RDP3 software (Table 3). In four out of eight analyzed genes significant evidence of recombination (P<0.05) was detected. However, the predicted recombination events have occurred in the orthologous DUXY copies of human and chimpanzee. This indicates that both events took place before the human-chimpanzee split and therefore do not influence the tree topology.

**Table 3:** Summary of recombination signal detection analysis performed by RDP3.

Event	Breakpoint positions					Detection Methods								
	Begin	End	Recombinant Sequence(s)	Minor Parental Sequence(s)	Major Parental Sequence(s)	RDP	GENE-CONV	Bootscan	Maxchi	Chimaera	SiSscan	PhylPro	LARD	3Seq
1	890	971	HSA_DUXY3	PTR_DUXY1	Unknown	ND	ND	ND	0.0306*	0.0535	ND	ND	ND	ND
			PTR_DUXY3											
2	420	841	HSA_DUXY1	Unknown	PTR_DUXY4	ND	ND	ND	0.6470	0.3635	0.0246*	ND	ND	0.0139*
			PTR_DUXY1											

Minor Parent = Parent contributing the smaller fraction of sequence.

Major Parent = Parent contributing the larger fraction of sequence.

Unknown = Only one parent and a recombinant need be in the alignment for a recombination event to be detectable.

ND = No signal detected.

* Significant recombination signals (P<0.05).

## Discussion

The human DUXY gene locus comprises four members of the *DUX4*-related gene family [8]. Our comparative FISH mapping of the DUXY genes in the hominoids clearly demonstrates that the gibbon Y chromosome was the first primate Y chromosome which acquired members of this gene family. In addition to the Y-chromosomal signal, the white-cheeked crested gibbon only presents signals in the middle of both chromosome 9 arms and the short arm of acrocentric chromosome 24 (Figure 3f; Table 1). No signals were detected on metaphase chromosomes of the rhesus macaque (data not shown). Nevertheless, it should be noted that *in silico* analyses of the rhesus macaque whole genome assembly detected sequences paralogous to the human DUXY genes. This may indicate the presence of evolutionarily more distantly related (rapidly evolving) paralogs in this species which escaped FISH detection with the human Y-derived cosmids. Recently it has been shown that *DUX4* orthologs are present in the rhesus macaque and the common marmoset [20]. However, despite the sequencing of the macaque Y chromosome has progressed considerably, no DUXY genes were detected.

In comparison to the gibbon, the great apes show an enormous increase and a more widespread genome distribution of DUXY paralogs. This increase does not coincide with the burst of duplication events that occurred approximately 15–25 million years ago, roughly correlating with the divergence of the Old World monkeys and hominoids [28]. By contrast, the slighter increase of DUXY paralogs towards the human coincides with the major burst of pericentromeric duplication activity that took place at the divergence time of the African great ape and human species (5 to 7 MYA [29]). Although the majority of the DUXY signals can be found at orthologous locations in great apes and human, there are several differences in the distribution pattern of DUXY paralogs reflecting lineage-specific gain and/or loss of *DUX4*-related gene family members (Figure 3, Table 1). Thereby, four regions attract particular attention (9p11.2; 2p11.1 and 2q21; 16cen) as they have been implicated to play a role in the evolution of hominids [30]. DUXY paralogs were detected exclusively in the pericentromeric region of human and gorilla chromosome 9, a region prone to pericentric inversions thereby altering the structural morphology of chromosome 9 twice during great ape and human evolution [30]–[33]. The orthologous centromeric regions of chromosome 2 (human) and chromosome 2A (pygmy chimpanzee and orangutan) [34] show DUXY paralogs, whereas no DUXY paralogs were detected at orthologous gorilla and common chimpanzee sites. Only the orangutan shows DUXY paralogs in the centromeric region of chromosome 2B, which corresponds to the ancestral centromere situated in human 2q21 [34]–[36]. The centromeric location of DUXY paralogs on human chromosome 16 reflects the ancestral chromosomal situation [37] and the differing DUXY paralog locations on the short arm of chromosome 16 (16p13) in chimpanzees and gorilla indicate the occurrence of species-specific pericentromeric inversions. All four examples provide clear evidence of the variability of DUXY-paralogous sequences in regions of the great ape and human genome affected by genomic instabilty.

The most prominent difference in the chromosomal distribution pattern of DUXY paralogs was detected on the hominoid Y chromosomes. All African great apes show a tripartite location of DUXY paralogs, with the distribution pattern being unique to each species Y chromosome. Even the most closely related chimpanzee species show distinct differences indicating the rapid and unconstrained evolution of the male-specific region of the Y chromosome (MSY) with respect to sequence content and structural organization.

Taken together, although our comparative mapping approach demonstrates the genome-wide distribution of DUXY paralogs, we noted clustering near centromeres and telomeres. These genomic regions are known to be enriched for interchromosomal duplications [38]. Due to the close relationship between the DUXY genes and the *DUX4* gene [8] it was not surprising to find DUXY paralogs near telomeric locations as all the primate *DUX4*-containing repeats were found in such genomic regions [39], [40]. Nevertheless, the substantial number of DUXY paralogs detected in pericentromeric regions points to an evolutionarily divergent complement of *DUX4*-related gene family members.

We also determined the basic structural organization of the orthologous DUXY loci in human and common chimpanzee. However, it should be noted that FISH experiments revealed the existence of two yet unsequenced paralogous DUXY loci on the common chimpanzee Y chromosome. The working draft sequences of three *DUX4*-related gene containing chimpanzee Y-chromosomal BAC clones might correspond to these loci. Interestingly, the size of the human locus is roughly five times smaller than the size of the orthologous chimpanzee locus. In each locus four DUXY genes were found to be arranged in a head-to-tail fashion unequally spaced by beta-satellite structures. Moreover, both DUXY loci are enframed by beta-satellite blocks demarcating them from the genomic environment. To unravel the evolutionary process forming the DUXY locus, we comparatively analyzed the putative DUXY gene ORFs and the beta-satellites of human and chimpanzee as well. The founding member of the DUX gene family (*DUX4* [11]) encodes a conserved protein originating from a retrotransposed copy of an intron-containing DUX gene containing two homeobox sequences [20]. All four human DUXY genes lack the potential to encode the complete second homeodomain [8]. Similarly, none of the chimpanzee DUXY gene copies encode a second homeodomain. One of the most intriguing features among the human DUXY gene copies was the detection of a 1-bp deletion in the putative DUXY1 ORF giving rise to a strikingly different carboxy terminus. The same 1-bp deletion was found in PTR *DUXY1* indicating that it was already present in the common ancestor of human and chimpanzees. The result of such an apparent frameshift would be an increase in both synonymous and nonsynonymous rates in each species. However, the underrepresentation of particularly nonsynonymous substitutions in the carboxy terminal part of the putative ORF common to both DUXY1 orthologs argues against this assumption. Nevertheless, the standard *d*_N_ */d*_S_ ratio of 2.14 indicated recent positive selection in the human lineage. Chimpanzee *DUXY2–4* gene copies show an identical stop codon (TGA) at codon 21 of the putative ORF, whereas all orthologous human copies do not show a stop codon at this position. Surprisingly, each of the human *DUXY2–4* gene copies show a unique nucleotide sequence at this codon position. In fact, all 4 human DUXY genes encode amino acids of different biochemical properties at codon 21. However, to what extent the absence of recombination, such as in the non-recombining region of the Y chromosome, influences the efficacy of selection in primates is still under debate [41]–[43].

Owing to the short stretch of preserved amino acid sequences in three chimpanzee DUXY gene copies, we performed the phylogenetic analyses on the nucleotide sequences of all human and chimpanzee DUXY genes as delimited by the ORF of HSA DUXY1. The phylogenetic reconstruction showed unequivocal paired assignment of the orthologous copies. All branching points were strongly supported using ML bootstrap analysis for nucleotide data sets, with the exception of the *DUXY2–4* relationship. Therefore, the resulting tree topology clearly argues for the existence of all DUXY genes prior to the human/chimpanzee split. To evaluate the impact of gene conversion on the tree topology of the DUXY locus, we conducted a recombination detection analysis with the same set of sequences used for phylogenetic reconstruction. The RDP3 analysis detected two significant recombination events, each within the orthologous human and chimpanzee *DUXY1* and *DUXY3* gene copies. In both instances, orthologous copies seem to be recombinant due to a gene conversion event between an unknown DUXY paralog and a chimpanzee *DUXY* (PTR *DUXY1*; PTR *DUXY4*) gene copy. Nevertheless, it should be noted that none of the human DUXY genes was identified as a parental nucleotide sequence, which might indicate that the human DUXY genes are more rapidly diverging than their chimpanzee counterparts. Taken together, the amino acid alignment survey, phylogenetic reconstruction, and recombination detection analyses do not only show the presence of the basic structural organization consisting of four DUXY gene copies in an ancestor of human and chimpanzees, but they also strengthen the *DUXY1* gene to be rated as the prototype of the Y-chromosomal DUX genes. Altogether, these data favour a mixed process of concerted and birth-and-death evolution acting on the DUXY gene family within the hominids [44].

Sequence analysis of the regions separating and enclosing the DUXY genes identified a complex genomic architecture of beta-satellite repeats. Five regions of beta-satellite repeat sequences were identified in human and chimpanzee as well. All of them were composed of a basic 68 bp chromosome Y beta-satellite monomer [45] organized into tandem repeats. These basic structural properties are highly reminiscent of previous reports on the organization of beta-satellite repeat regions in proximity to the centromere [45], [46], but also present striking similarities to the zinc finger gene cluster on chromosome 19p12 [47]. In four out of five orthologous beta-satellite blocks (I, II, IV, V) the size of the human repeat was equal to or moderately larger than its chimpanzee counterpart. Comparable to the human beta-satellite block III (∼4 kb), the orthologous chimpanzee repeat block has experienced a major expansion (∼145 kb). This unique property is based on the temporally independent development of two higher-order repeat structures ranging in length from 45 to 98 kb, one of them originated from the amplification of a defined monomer of the former one. Unequal crossing-over events between sister chromatids during meiosis or saltatory replication might be the mechanisms accounting for the size differences between the orthologous beta-satellite repeat regions [48]. A remarkable sequence conservation was observed for the orthologous monomers in each of the orthologous beta-satellite blocks (Table 2), similar to orthologous monomeric alpha-satellites, which diverge less rapidly than the higher-order alpha-satellites [49]. The majority of these beta-satellite monomers contain the highly conserved nucleotide block GATCAGTGC which has been proposed to function as a protein-binding site for this repeat [50], [51] and thus might be predicted to be subject to selection.

Pericentromeric regions of human chromosomes have been created by duplicative transposition of euchromatic segments that have invaded centromeric transition regions over the past 35–40 million years of evolution [38]. As the beta-satellites seem to have been amplified along with the DUXY genes, the most obvious explanation is that both were part of an ancestral DUX gene cassette that became duplicated and/or transposed. The genomic region mediating the transfer of the ancestral DUX cassette to the primate Y chromosome during evolution might have been the nucleolus organizer region (NOR) as a close genomic association of DUX genes with beta-satellite repeats and rDNA has been shown to exist on the short arms of acrocentric chromosomes [40]. Moreover, FISH experiments on the white-cheeked crested gibbon showed the presence of DUXY paralogs in the Y-chromosomal NOR.

We propose a model in which the hominoid DUXY loci have most likely arisen by an evolutionary mechanism involving the transposition of an ancestral tandem array DUX gene cassette to the hominoid Y chromosome followed by lineage-specific chromosomal rearrangements that are rather common during speciation events. The subsequent reproductive isolation of the hominoid species allowed evolutionary forces to act separately on the DUXY loci thereby promoting the divergence of the four gene copies. As positive selection was shown to act on human *DUXY1*, the re-activation of the predicted coding potential of the human *DUXY2–4* genes might be explained by directional selection, too. Human and chimpanzee *DUXY1* may have retained the original function while only in human *DUXY2–4* acquired novel, evolutionary advantageous functions (neo-functionalisation [52]). Of course, other mechanisms such as relaxed purifying selection due to genetic drift or genetic hitchhiking [53 and references therein] due to sperm competition might also provide a suitable explanation for this observation. To explore the basis of this distinctive feature, comparative sequencing of the hominoid DUXY loci in combination with comparative expression profiling will be required. It will be interesting to examine the hominoid DUXY loci to better understand the evolutionary events that shaped the distinct yet related molecular properties of these loci on different primate Y chromosomes.

In its current state, both the human and the common chimpanzee DUXY locus meet essential characteristics of ‘cores’ of human genome evolution [54]. They are flanked by large blocks of segmental duplications and carry presumably rapidly evolving genes. The failure to identify the human DUXY locus as a ‘core’ in [54] is easily explained by the absence of the DUXY locus containing contig in the human genome reference assembly analyzed in their study. Furthermore, no other similarly structured DUX locus is present in the current human genome reference assembly probably suggesting the Y-specific development of this ‘core’. Future studies will be needed to see if the primate DUXY locus is prone to microdeletions and microduplications and therefore subject to within-species and between-species copy number variation among different primates and the human population.

### Ethics Statement

The non-human primate blood samples were obtained by zoo-physicians only during anesthesia of the animals on important medical attendance. All treatments followed the guidelines of the relevant local Ethics Committees (Zoological Garden Wilhelma, Stuttgart; Zoological Garden Duisburg; German Primate Research Center, Göttingen) on Research involving non-human primate subjects.

## Materials and Methods

### Identification of DUXY-containing cosmid clones

DUXY-specific primers (F: 5′-CTTTCGCCTGCCTTCTTG-3′; R: 5′-CGACAACTTCGGACAGCA-3′) were used to amplify a fragment of 281 bp from the DUXY-locus containing BAC clone RP11-886I11 (AC134882). The band was excised, purified and ^32^P-labeled according to Feinberg and Vogelstein [55]. For detection of DUXY-positive cosmid clones, filters carrying the Y-chromosome specific LLNL library LL0YNC03″M″ were hybridized with the ^32^P-labeled fragment at 1×10^6^ cpm/ml. Pre- and hybridization were carried out in 50% formamide, 2× SSC, 50 mM Na_2_HPO_4_, 1 mM EDTA, 50 µg/ml sheared salmon sperm DNA, 10× Denhardt's reagent, and 1% SDS at 42°C. Filters were washed in 1×SSC/1%SDS at 65°C for 1 h, exposed for 12 h to X-ray film at RT and positive cosmid colonies determined.

Cosmids and BAC RP11-886I11 were isolated according to the manufacturer's conditions (QIAgen-tip 100; QIAgen GmbH), digested with NcoI (New England Biolabs, Inc.), fractionated on a 1% agarose gel and transferred to a Hybond-N^+^-membrane (Amersham International plc.). Southern blot hybridization was carried out at high stringency conditions in a heparin hybridization buffer [56], the filter was washed at 0.1× SSC/1%SDS at 65°C and the blot exposed to X-ray film for 30 min at RT.

### Chromosome preparation

Standard chromosome preparations were applied to peripheral lymphocyte cultures [57] of male individuals of human (*Homo sapiens*, HSA), chimpanzee (*Pan troglodytes*; PTR), bonobo (*Pan paniscus*, PPA), gorilla (*Gorilla gorilla*, GGO), Bornean and Sumatran orang-utan (*Pongo pygmaeus*, PPY), white-cheeked crested gibbon (*Nomascus leucogenys*; NLE), and rhesus macaque (*Macaca mulatta*; MMU). For the non-human lymphocyte cultures, the method varied only in the time of exposition to colcemide, i.e. 2 h instead of 1 h for human lymphocyte cultures. Heparinized blood samples for chimpanzee, bonobo, and gorilla were provided by Dr. W Rietschel of the Wilhelma Zoo Stuttgart, Germany. Orangutan blood samples were obtained from Dr. MG Hartmann of the Zoo Duisburg and the rhesus macaque blood samples were provided by the German Primate Research Center in Göttingen. Metaphase preparations of the gibbon species *Nomascus leucogenys* were obtained from a lymphoblastoid cell line, kindly provided by S. Müller, Munich. Metaphases of different individuals were used for FISH analysis. A minimum of 15 metaphases were scored per individual.

### Fluorescence in situ hybridization (FISH)

Prior to FISH, the slides were treated with RNase followed by pepsin digestion as described previously [58]. FISH followed the method described in [57]. Chromosome *in situ* suppression was applied to the following genomic clones: the IL3RA-containing cosmid ICRFc104E0238 [59], the SHOX-containing cosmid LL0YNC03″M″-34F05 [60] and the DUXY-containing cosmids LL0YNC03 ″M″-38D05, -39H03, -70B12, and -118E07. The plasmid pMr100 [61] served as a marker of the NOR and whole chromosome paints (Mariano Rocchi, Bari, Italy; Johannes Wienberg, Munich, Germany) were used to identify human-gibbon syntenic regions. After FISH the slides were counterstained with DAPI (0.14 µg/ml) and mounted in Vectashield (Vector Laboratories).

### Fluorescence microscopy and imaging

Metaphase preparations were evaluated using a Zeiss Axiophot epifluorescence microscope equipped with single-band pass filters for excitation of red, green, and blue (Chroma Technologies, Brattleboro, VT). During exposures, only excitation filters were changed allowing for pixel-shift-free image recording. Images of high magnification and resolution were obtained using a black-and-white CCD camera (Photometrics Kodak KAF 1400; Kodak, Tucson, AZ) connected to the Axiophot. Camera control and digital image acquisition involved the use of an Apple Macintosh Quadra 950 computer.

### Sequence dataset compilation

The nucleotide sequence of the complete ORF of HSA DUXY1, previously shown to reside in the human BAC clone RP11-886I11 (AC134882), was used as a reference sequence to identify the paralogous and orthologous DUXY sequences in the chimpanzee BAC clone CH251-549O17 (AC185324). Sequences were extracted from GenBank and aligned using CLUSTALW 2.0 [22]. The completed alignment was manually trimmed and edited in MEGA 4.0 [62].

To identify the stretches of beta-satellite monomers within both BAC clones, we RepeatMasked the complete sequences of the human and chimpanzee DUXY loci. We used the basic repeat unit of the beta-satellite (X65994) to isolate 269 monomers from the human BAC clone and 2237 monomers from the chimpanzee BAC clone. The 2506 beta-satellite monomers were aligned by CLUSTALW 2.0 [22] and manually examined and edited using MEGA 4.0 [62]. A sequence identity matrix was generated by BioEdit 7.0.9 [63] and visualized by HeatMap Builder 1.1 [64].

### Phylogenetic analysis

The complete edited alignment of all human and chimpanzee DUXY sequences was used in MODELTEST [65] to determine the model of nucleotide evolution best fitting the sequences. ML model parameters chosen by MODELTEST were used in a heuristic tree search using PAUP*4.0b [66] with the PaupUp graphical interface [67]. Nexus formatted tree files were edited in TreeView [68]. A standard McDonald-Kreitman test [69] was performed in DnaSP [70] for *DUXY1* orthologs to compare the numbers of nonsynonymous and synonymous substitutions.

To generate the beta-satellite monomer phylogenetic tree, we used the CLUSTALW alignment of all monomers from the human and chimpanzee DUXY loci. The 102 multimer higher-order repeat (HOR) array identified in chimpanzee satellite region III was subdivided in its multimers and a multimer consensus sequence calculated by MEME analysis [71]. The monomers extracted from the multimer consensus sequence were used to replace all distinct monomers of the 102 multimer HOR in the CLUSTALW alignment file. Phylogenetic reconstruction of the beta-satllite monomers was carried out by MEGA 4.0 [62]. Neighbor-joining methods were used with pairwise deletion parameters and 1000 bootstrap iterations.

### Recombination detection

We screened all HSA and PTR DUXY sequences in order to detect potential recombination sequences, and to identify their likely parent sequences and locate the possible recombination breakpoints with RDP3 (Recombination detection program Vers. 3.32). The RDP3 software implements exploratory methods (RDP [72], GENECONV [73], BOOTSCAN [74], MAXIMUM CHI SQUARE [75], CHIMAERA [76], 3SEQ [77], and SISTERSCAN [78]) as well as supplementary methods (LARD [79] and PHYLPRO [80]. The general settings for all methods executed in RDP3 were as follows: Sequences were considered to be linear, the p-value cutoff was set to 0.05, the standard Bonferroni correction was used, consensus daughters were identified and breakpoints polished.

## Supporting Information

Figure S1 Pairwise comparisons of monomers of orthologous human and common chimpanzee beta-satellite blocks were calculated and percent identity scores visualized according to the color scale. The species origin of beta-satellite monomers is shown at the top of each figure in black (HSA) and white (PTR) letters. (A, B, C) Heat maps illustrating the pairwise comparisons for monomers from beta-satellite regions I, II, and IV.
(7.76 MB TIF)

Figure S2 Phylogenetic tree of beta-satellites from the human and common chimpanzee DUXY locus. Neighbor-joining methods were used to generate the phylogenetic tree containing monomeric beta-satellites from the orthologous beta-satellite repeat regions. Additionally, beta-satellites from the HOR array consisting of 45 multimeric repeat units from common chimpanzee beta-satellite region III were included. The resulting tree consists of 976 monomers. The colour key at the bottom of the figure indicates the species and beta-satellite region origin from monomeric and higher-order beta-satellites
(0.76 MB TIF)

Text S1 (0.02 MB DOC)
