# Supplementary figures and images for: Complex Evolution of a Y-Chromosomal Double Homeobox 4 (DUX4)-Related Gene Family in Hominoids
Source: PLoS One. 2009 Apr 30;4(4):e5288. doi: 10.1371/journal.pone.0005288 (PMC2671837; doi:10.1371/journal.pone.0005288)

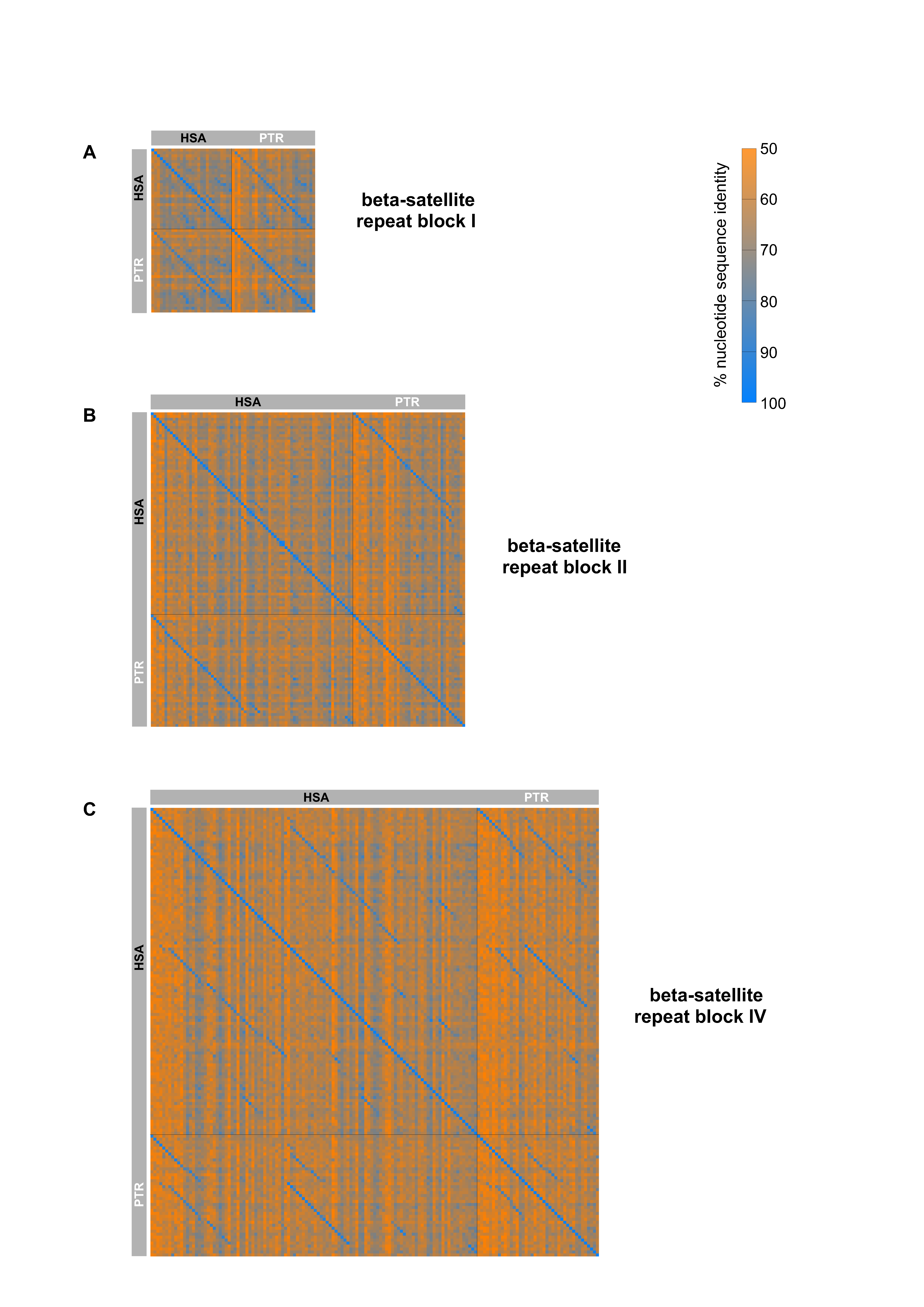

Supplement: Figure S1 — Pairwise comparisons of monomers of orthologous human and common chimpanzee beta-satellite blocks were calculated and percent identity scores visualized according to the color scale. The species origin of beta-satellite monomers is shown at the top of each figure in black (HSA) and white (PTR) letters. (A, B, C) Heat maps illustrating the pairwise comparisons for monomers from beta-satellite regions I, II, and IV. (7.76 MB TIF) [file pone.0005288.s001.tif]

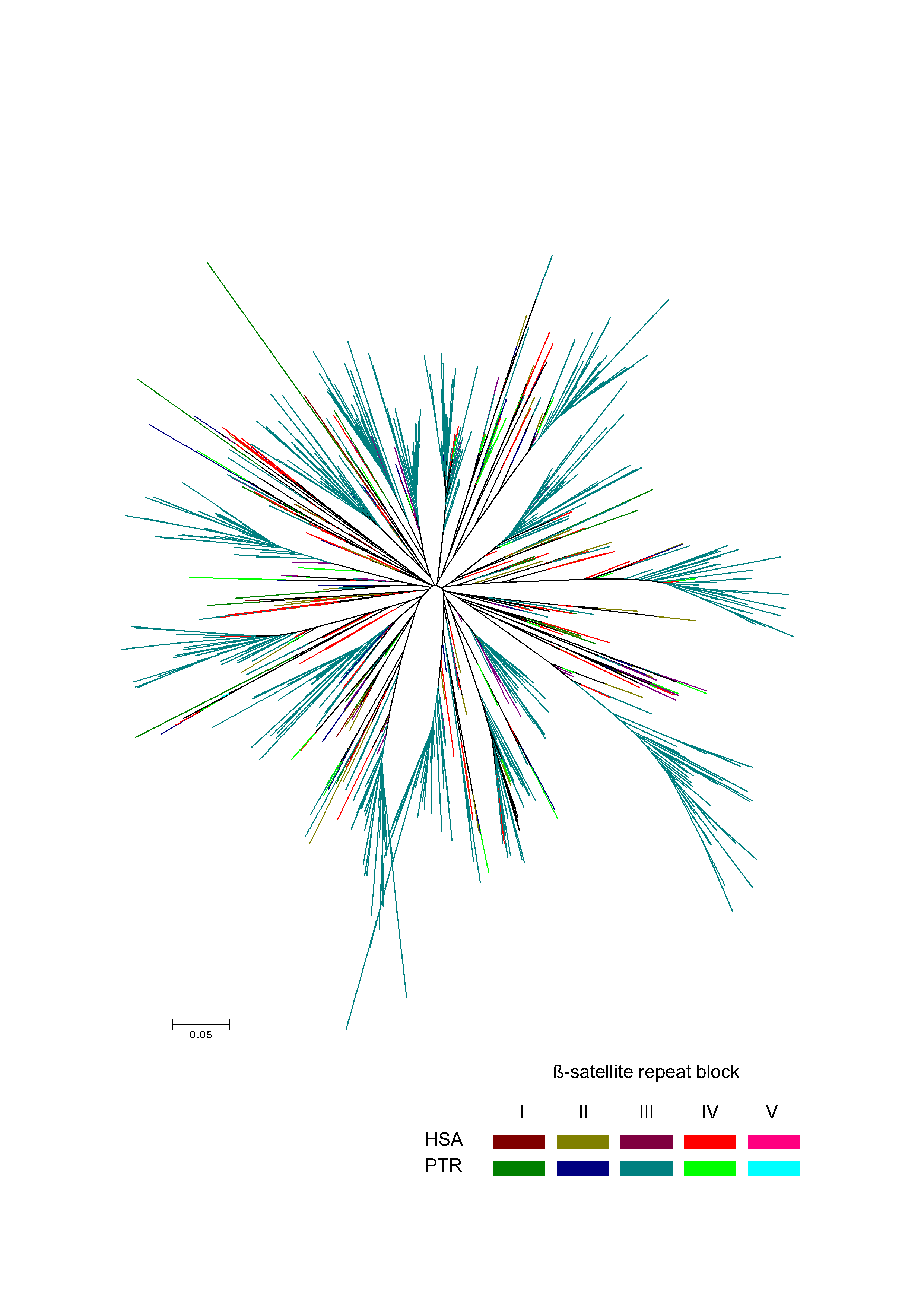

Supplement: Figure S2 — Phylogenetic tree of beta-satellites from the human and common chimpanzee DUXY locus. Neighbor-joining methods were used to generate the phylogenetic tree containing monomeric beta-satellites from the orthologous beta-satellite repeat regions. Additionally, beta-satellites from the HOR array consisting of 45 multimeric repeat units from common chimpanzee beta-satellite region III were included. The resulting tree consists of 976 monomers. The colour key at the bottom of the figure indicates the species and beta-satellite region origin from monomeric and higher-order beta-satellites (0.76 MB TIF) [file pone.0005288.s002.tif]
