# Supplementary material for: Complex Evolution of a Y-Chromosomal Double Homeobox 4 (DUX4)-Related Gene Family in Hominoids
Source: PLoS One. 2009 Apr 30;4(4):e5288. doi: 10.1371/journal.pone.0005288 (PMC2671837; doi:10.1371/journal.pone.0005288)
Supplement: Text S1 — (0.02 MB DOC) [file pone.0005288.s003.doc]

**Comparative analysis of human and chimpanzee beta-satellite repeat block III**

The monomeric percent identity within chimpanzee beta-satellite block III (80,9%) was significantly higher than that in the orthologous human region (73.4%). Sequence identity matrix analysis of the chimpanzee beta-satellite block III identified two distinct classes of higher-order repeat (HOR) arrays. One HOR array consists of 102 multimeric repeat units, each basically made up of 15 monomers with a mean percent identity of 98.4 %, a value typically found in HOR arrays of alpha-satellites [1,2]. After substituting all multimers of this repeat unit by a multimer consensus sequence, the mean percent identity of the chimpanzee beta-satellite block III decreased to 71.9%. The second HOR array consists of 45 multimeric repeat units, each basically made up of 15 monomers with a mean percent identity of 73.5%. This HOR array was disrupted in multimer 10 by the development of the former repeat unit.

We also used neighbor-joining methods to examine the phylogenetic relationship among the human and chimpanzee beta-satellite repeat blocks. For this purpose, we replaced the 102 multimer repeat unit by a multimer consensus sequence. The resulting phylogenetic tree (Fig. S2) indicates, as expected, 15 sub-clades depicting the 15 beta-satellite monomers forming the more diverged 45 multimer repeat unit. All eight clades do not only contain beta-satellite monomers from orthologous repeat regions, but also from different repeat regions indicating that the monomers can be traced back to one founding beta-satellite repeat block.

# References

1 Warburton PE, Willard HF (1995) Interhomologue sequence variation of alpha satellite DNA from human chromosome 17: evidence for concerted evolution along haplotypic lineages. J Mol Evol 41: 1006-1015.

2 Warburton PE, Haaf T, Gosden J, Lawson D, Willard HF (1996) Characterization of a chromosome-specific chimpanzee alpha satellite subset: evolutionary relationship to subsets on human chromosomes. Genomics 33: 220-228.

**Figure Legends**

**Figure S1:** Pairwise comparisons of monomers of orthologous human and common chimpanzee beta-satellite blocks were calculated and percent identity scores visualized according to the color scale. The species origin of beta-satellite monomers is shown at the top of each figure in black (HSA) and white (PTR) letters. ***(A, B, C)*** Heat maps illustrating the pairwise comparisons for monomers from beta-satellite regions I, II, and IV.

**Figure S2:** Phylogenetic tree of beta-satellites from the human and common chimpanzee DUXY locus. Neighbor-joining methods were used to generate the phylogenetic tree containing monomeric beta-satellites from the orthologous beta-satellite repeat regions. Additionally, beta-satellites from the HOR array consisting of 45 multimeric repeat units from common chimpanzee beta-satellite region III were included. The resulting tree consists of 976 monomers. The colour key at the bottom of the figure indicates the species and beta-satellite region origin from monomeric and higher-order beta-satellites.
